# Real-world analysis of the use of lenvatinib in differentiated thyroid cancers

**DOI:** 10.3332/ecancer.2023.1500

**Published:** 2023-01-30

**Authors:** Zoya Peelay, Deevyashali Parekh, Vijay M Patil, Vanita Noronha, Nandini Menon, Kumar Prabhash

**Affiliations:** Department of Medical Oncology, Tata Memorial Hospital, HBNI, Parel, Mumbai 400012, India

**Keywords:** lenvatinib, thyroid cancer, papillary, follicular, radioiodine refractory, dedifferentiated

## Abstract

**Introduction:**

Lenvatinib is one of the approved treatments for radioiodine-refractory differentiated thyroid cancers. However, there is very limited data from India on real-world efficacy and adverse events of Lenvatinib and hence this analysis was performed.

**Methods:**

This was a retrospective analysis in which patients of radioiodine-refractory differentiated thyroid cancer as per the SELECT study criteria, who received lenvatinib, were selected for the study over the last 4 years. The baseline demographic characteristics, adverse events of lenvatinib, the date of progression and the date of overall survival (OS) were extracted from the electronic medical records of Tata Memorial Hospital. SPSS version 20 was used for analysis.

**Results:**

The median starting dose of lenvatinib was 20 mg. Fifteen events for progression had occurred and the median progression-free survival (PFS) was 12.2 months [95% CI: 4.4–not available (NA)]. The events for OS analysis were 12. The median OS was 35.3 months (95% CI: 11.4–NA). There was no impact on starting dose on PFS or OS.

**Conclusion:**

The real-world data of Lenvatinib suggest a lot of variability in the starting dose. In spite of this variability, the response rates and OS are similar to that noted in pivotal study. This suggests a case for need for more studies evaluating lower doses of Lenvatinib.

## Introduction

Thyroid cancers are a relatively uncommon malignancy in India; however, amongst them, differentiated thyroid cancers are one of the commonest malignancies. Differentiated thyroid cancers are treated with surgery followed by radioiodine therapy as per the risk stratification. In spite of this treatment, nearly two third of patients will become radioiodine refractory over their lifetime. When the patients become radioiodine refractory, treatment options are very limited. Lenvatinib was not available in India until recently and hence these patients were treated with sorafenib. Lenvatinib became available in India in 2018 and has been used frequently since then.

Lenvatinib is a multi-kinase inhibitor. Different doses of lenvatinib are used in different cancer settings and there remain concerns with respect to adverse events of lenvatinib. Grade 3 and above adverse events are seen in more than 50% of patients. Thus, frequent dose modifications are required. There is very limited data from India about the efficacy, compliance and side effects of lenvatinib, hence this study was contemplated. Patients who have radioiodine refractory thyroid cancers and are treated with lenvatinib over the past 4 years were included in the study. The primary objective was to study the efficacy of lenvatinib and the secondary objective was to study the adverse events and compliance in dedifferentiated thyroid cancers.

## Methods

### Selection of patients

The Head and Neck Medical oncology unit of Tata Memorial Center maintains a prospective database of all the patients undergoing chemotherapy under their care. Patients were selected from this database for the current analysis. The criteria to select these patients were as follows:

1. Adult patients of age ≥ 18 years
2. Diagnosed with thyroid cancer
3. Treated with lenvatinib
4. Time period from January 2018 to December 2021
5. The patients who satisfied all the above-mentioned four criteria were selected.

### Data collection

The data regarding baseline characteristics, previous treatment details, lenvatinib administration, compliance to it, adverse events on it, response, date of progression, site of progression, date of death and status at last follow-up were curated from the prospective database and electronic medical records.

### Endpoint definitions

Response assessment was done as per RECIST 1.1 criteria and the adverse events were recorded at every visit based on Common Terminology Criteria for Adverse Events (CTCAE) version 5.0. Progression-free survival (PFS) was defined as time from start of lenvatinib to progression or death whichever occurred earlier. Overall survival (OS) was defined as the time from the start of lenvatinib to death.

### Statistical analysis

Descriptive statistics were performed. The Kaplan–Meier method was used for the estimation of PFS and OS. The impact of starting dose of lenvatinib on PFS and OS was estimated using Cox regression analysis. A *p*-value of 0.05 was considered as significant.

## Results

### Baseline characteristics

We identified 27 patients who had received Lenvatinib. The baseline characteristics are provided in Table 1. All patients had metastatic disease and were radioiodine refractory.

### Compliance

**Figure 1.**
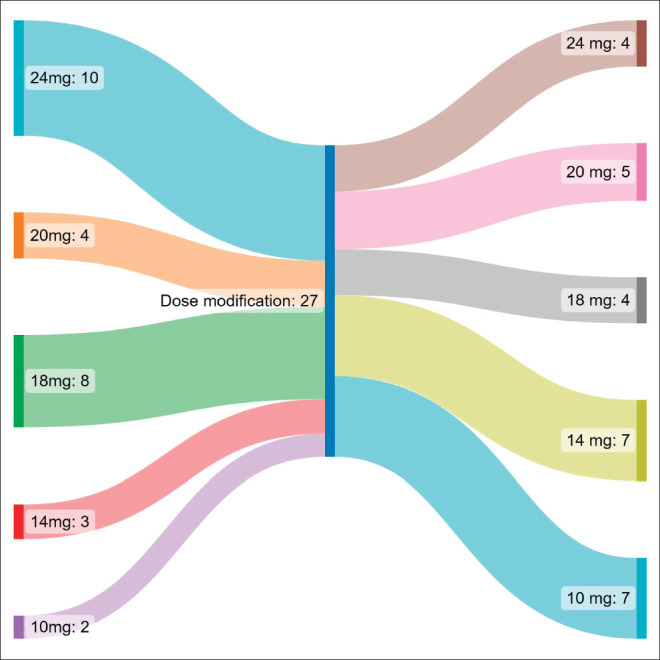
Sankey diagram representing the change in dose in patients taking Lenvatinib.

Table 2 shows the adverse event profile. The starting dose was 24 mg in 10 (37%), 20 mg in 4 (14.8%), 18 mg in 8 (29.6%), 14 mg in 3 (11.1%) and 10 mg in 2 (7.4%) patients. The median starting dose of lenvatinib was 20 mg. The dose escalation and de-escalation was performed in three patients, two of them had a starting dose of 10 mg which was escalated to 14 mg and in one patient the dose was increased from 20 mg to 24 mg. The dose interruption had to be done in 17 patients, the dose reduction had to be done in 15 patients and the number of interruptions was single and multiple in 5 and 12 patients, respectively. The dose settled was 24 mg in 4 (14.8%), 20 mg in 5 (18.5%), 18 mg in 4 (14.8%), 14 mg in 7 (25.9%) and 10 mg in 7 (25.9%) patients (Figure 1). Lenvatinib had to be stopped due to toxicity in four patients (14.8%). Lenvatinib had to be stopped due to toxicity in four patients (14.8%), recurrent proteinuria in 2 (7.4%) patients and hand foot syndrome (HFS) in 2 (7.4%) patients.

### Outcome

**Figure 2.**
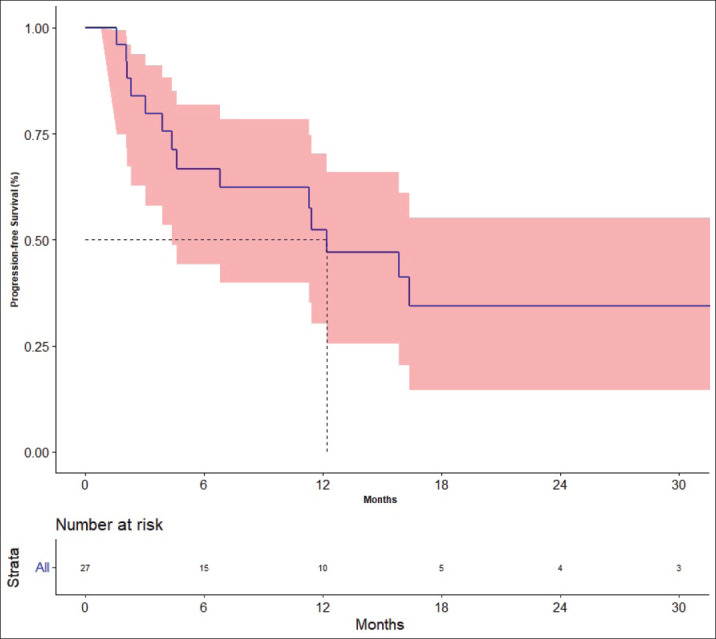
Progression-free survival.

**Figure 3.**
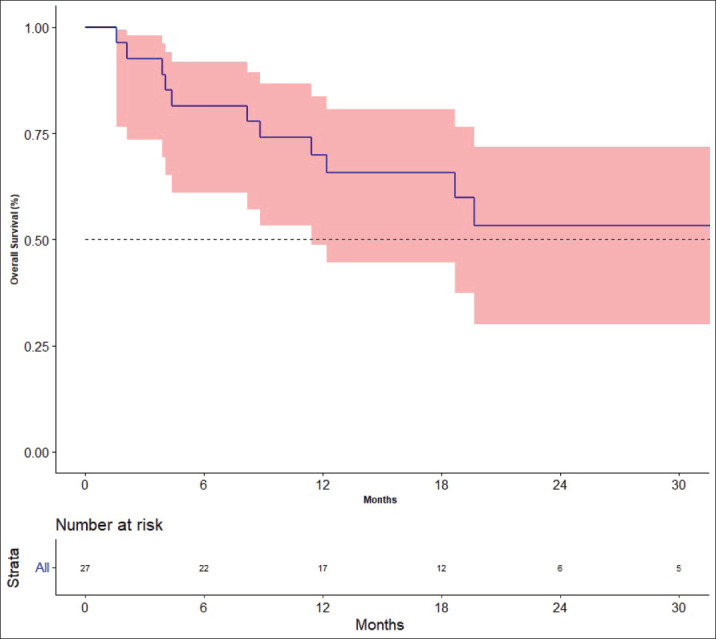
Overall survival.

The response rates are shown in Table 3. The best response among the four patients previously treated with lenvatinib was partial response in one patient and stable disease (SD) in three patients. The median follow-up was 19.7 months. Fifteen events for progression had occurred and the median PFS was 12.2 months [95% CI: 4.4–not available (NA)]. The 1- and 2-year PFS was 52.2% (95% CI: 30.2–70.4) and 34.3% (95%CI: 14.5–55.2), respectively (Figure 2). There was no impact of starting dose on PFS. (Hazard ratio: 1.020; 95% CI: 0.890–1.170, *p* = 0.764). The events for OS analysis were 12. The median OS was 35.3 months (95% CI: 11.4–NA). The 1- and 3-year OS was 70.0% (95% CI: 48.7–83.7) and 29.9% (95% CI: 19.6–69.1), respectively (Figure 3). There was no impact of starting dose on OS (Hazard ratio: 1.090; 95% CI: 0.930–1.280, *p* = 0.303). With regard to subjects receiving treatment without a surgical thyroidectomy due to inoperable cancer, the outcomes in neoadjuvant chemotherapy (NACT) lenvatinib for these patients were 50% response rate and Median OS was 25.56 months (95% CI: 7.9–43.23).

## Discussion

To the best of authors’ knowledge, this is one of the first such sets of data from India looking at real-world applicability of lenvatinib in thyroid cancer. Lenvatinib was not available in India for a long period of time. The use of lenvatinib has been associated with a high amount of adverse events [1–7]. For the use of Lenvatinib in multiple other sites, the doses used are variable ranging from 10 mg to 24 mg [1–7]. However, because of the adverse event profile, there was a concern if trial data could be applicable in a real-world setting [8]. This is the novelty of this study.

Compared to the SELECT trial [9], which also studied the efficacy of Lenvatinib, our study reported a slightly lower median PFS (12.2 versus 18.3 months). The overall response rates were similar, 62.9% in our study versus 64.7% in SELECT. This difference may be due to certain key differences between the two studies. 66.7% of the patients in our study were Eastern Cooperative Oncology Group (ECOG) performance status (PS) 0-1 while 95% of patients in SELECT were ECOG PS 0-1. This might explain the lower PFS from our study.

The adverse event profile was similar in both studies. However, this was in spite of a lower dose of Lenvatinib started in 63% (17 out of 27 patients). The dose interruptions and dose reductions with Lenvatinib in literature are in the tune of 50%–97% [10–15]. Physician discretion was used in our study for the starting dose and depending on tolerances the dose was either escalated or de-escalated as was done in other similar studies [16, 17]. The use of 24 mg starting dose is associated with a survival benefit in literature [9, 18, 19], as is the lack of dose interruptions [20]. Hence, it is important to select a starting dose of 24 mg for patients. In our study, ten patients were started with a 24 mg dose and out of them, four could continue on the dose. Our study suggests that if an effective dose escalation and de-escalation is used, the starting dose of Lenvatinib is unlikely to impact outcomes.

The study has a few inherent limitations. This was a retrospective study in a single centre. Furthermore, our sample size is relatively small to draw robust, reliable conclusions.

## Conclusion

The real-world data of Lenvatinib suggest a lot of variability in the starting dose. In spite of this variability, the response rates and OS are similar to that noted in pivotal study. This suggests a case for need for more studies evaluating lower doses of Lenvatinib.

## Conflicts of interest

None.

## Financial declaration

No funding was obtained for this study.

## Figures and Tables

**Table 1.** Baseline characteristics of patients treated with lenvatinib. ECOG, Eastern Cooperative Oncology Group; PS, Performance status.

Variable	Value (*n* = 27)
Age – No. (%) Median Interquartile-range	56 45–61.0
Gender – No. (%) Male Female	12 (44.4) 15 (55.6)
ECOG PS – No. (%) PS0-1 PS2 PS3	18 (66.7) 4 (14.8) 5 (18.5)
Thyroid status – No. (%) Hypothyroid Euthyroid Hyperthyroid	9 (33.3) 11 (37.0) 8 (29.6)
Comorbidities Hypertension Diabetes mellitus Ischaemic heart disease	5 (18.5) 4 (14.8) 1 (3.7)
Pathology Papillary thyroid cancer Follicular thyroid cancer	17 (63) 10 (37)
Previous treatment Surgery Radioiodine Radiotherapy Sorafenib	19 (70.4) 13 (48.1) 8 (29.6) 4 (14.8)
Radioiodine refractory criteria Progressive disease (PD) within 12 months of treatment with Radioactive Iodine (RAI) Cumulative RAI dose of ≥600 millicurie (mCi) No radioiodine uptake Inoperable thyroid carcinoma	
Site of metastasis Lung Bone Liver Brain	19 (66.7) 12 (44.4) 5 (18.5) 2 (7.4)

**Table 2.** Adverse events due to lenvatinib and their grading. HFS, Hand foot syndrome.

Adverse event (%)	Grade				
	1	2	3	4	5
HFS	3 (11.1)	10 (37)	3 (11.1)	0	0
Proteinuria	3 (11.1)	4 (14.8)	4 (14.8)	0	0
Hypertension	9 (33.3)	0	0	0	0
Oral mucositis	6 (22.2)	4 (14.8)	1 (3.7)	0	0
Anaemia	1 (3.7)	3 (11.1)	0	0	0
Transaminitis	2 (7.4)	2 (7.4)	0	0	0
Diarrhoea	4 (14.8)	5 (18.5)	0	0	0
Fatigue	8 (29.6)	6 (22.2)	3 (11.1)	0	0
Myalgia	8 (29.6)	4 (14.8)	0	0	0
Neutropenia	1 (3.7)	0	0	0	0
Thrombocytopenia	2 (7.4)	0	1 (3.7)	0	0
Itching	1 (3.7)	0	0	0	0
Rash	1 (3.7)	0	0	0	0

**Table 3.** Response assessment from baseline till treatment completion.

Response – No(%)	*N* = 27
Response rate	17 (62.9)
Stable disease (SD)	7 (25.9)
Progressive disease (PD)	3 (11.1)
